# Polyamine Oxidases Play Various Roles in Plant Development and Abiotic Stress Tolerance

**DOI:** 10.3390/plants8060184

**Published:** 2019-06-21

**Authors:** Zhen Yu, Dongyu Jia, Taibo Liu

**Affiliations:** 1 State Key Laboratory for Conservation and Utilization of Subtropical Agro-Bioresources, Guangdong Provincial Key Laboratory of Protein Function and Regulation in Agricultural Organisms, College of Life Sciences, South China Agricultural University, Guangzhou 510642, China; yuzhen5500@163.com; 2 Department of Biology, Georgia Southern University, Statesboro, GA 30460-8042, USA; djia@georgiasouthern.edu

**Keywords:** back conversion pathway, polyamines, polyamine oxidase, polyamine catabolism, stress response, terminal catabolism pathway

## Abstract

Polyamines not only play roles in plant growth and development, but also adapt to environmental stresses. Polyamines can be oxidized by copper-containing diamine oxidases (CuAOs) and flavin-containing polyamine oxidases (PAOs). Two types of PAOs exist in the plant kingdom; one type catalyzes the back conversion (BC-type) pathway and the other catalyzes the terminal catabolism (TC-type) pathway. The catabolic features and biological functions of plant PAOs have been investigated in various plants in the past years. In this review, we focus on the advance of PAO studies in rice, Arabidopsis, and tomato, and other plant species.

## 1. Introduction

Polyamines (PAs) are aliphatic amines of small molecular mass that are involved in various biological processes [1,2]. The putrescine (Put), cadaverine (Cad), spermidine (Spd), spermine (Spm), and thermospermine (T-Spm) are the major plant PAs [1,2,3,4,5,6,7]. PAs play important roles in embryogenesis, cell division, organogenesis, flowering, programmed cell death (PCD), response to abiotic and biotic stresses, and so on [4,5,6,7,8,9,10,11,12,13,14,15,16,17,18,19,20,21,22,23,24,25,26,27,28,29,30,31,32,33].

The homeostasis of cellular PA levels, being well regulated by a dynamic balance of biosynthesis and catabolism, is most important for maintaining normal growth and development in plants. The PA biosynthetic pathway has been well elucidated [1,34,35], however, the PA catabolism pathway remains unclear in spite of more and more newly identified genes in this pathway in plants [4,36,37,38,39,40,41,42,43,44,45,46,47,48,49,50,51,52,53,54,55,56]. In this review, we summarized the advances of the polyamine oxidases’ (PAOs) roles in PA catabolism, plant development, and abiotic stress tolerance from rice, Arabidopsis, tomato, and other plant species.

## 2. PA Biosynthesis in Plants

**Figure 1:**
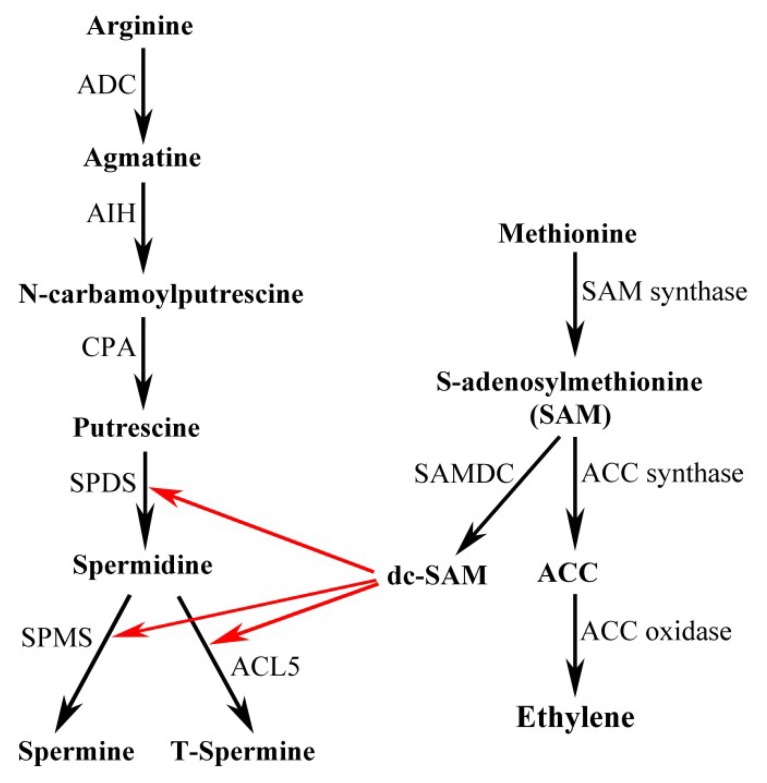
Polyamine biosynthesis pathway in *Arabidopsis thaliana*. ADC, arginine decarboxylase; AIH, agmatine iminohydrolase; CPA, *N*-carbamoylputrescine amidohydrolase; SPDS, Spd synthase; SPMS, Spm synthase; ACL5, ACAULIS5, T-Spm synthase; SAM, *S*-adenosylmethionine; SAMDC, *S*-adenosylmethionine decarboxylase; dcSAM, decarboxylated *S*-adenosylmethionine; ACC, 1-amino-cyclopropane-1-carboxylic-acid.

Plant PA biosynthesis is rather short, which starts mainly from arginine (Arg). The pathway is briefly shown in Figure 1 and is described as follows. Firstly, Arg is converted to Put via agmatine by three sequential reactions catalyzed by arginine decarboxylase (ADC, EC 4.1.1.19), agmatine iminohydrolase (AIH, EC 3.5.3.12), and *N*-carbamoylputrescine amidohydrolase (CPA, EC 3.5.1.53). Besides, some plants have the ornithine decarboxylase (ODC, EC 4.1.1.17) which catalyzes ornithine to Put directly [57], but Arabidopsis has only the ADC pathway because it lacks *ODC* genes. Secondly, the diamine Put is converted to triamine Spd by Spd synthase (SPDS, EC 2.5.1.16). Finally, Spd is further converted to Spm or T-Spm, two tetraamine isomers, by Spm synthase (SPMS, EC 2.5.1.22) and T-Spm synthase (ACAULIS5, abbreviated to ACL5), respectively [9,19,47,58]. An aminopropyl group is transferred from the decarboxylated *S*-adenosylmethionine (dcSAM) produced from methionine in two sequential reactions catalyzed by methionine adenosyltransferase and *S*-adenosylmethionine decarboxylase (SAMDC), respectively. These aminopropyl groups participate in the biochemical reaction of Spd, Spm, and T-Spm biosynthesis processes. Additionally, norspermidine (NorSpd) and norspermine (NorSpm), having been found as “uncommon PAs” due to their limited distribution in nature, are predicted to be synthesized either successively by each specific aminopropyl transferase (APT) or by a single APT with broad substrate specificity from 1,3-diaminopropane (1,3-DAP) [59].

## 3. PA Catabolism in Plants

PA biosynthetic pathways have been well investigated. In contrast, the knowledge on PA catabolism in plants is still fragmental though scholars reported some new findings in the past years. Two kinds of enzymes are involved in PA catabolism. Namely, one is a copper-dependent diamine oxidase (DAO, EC 1.4.3.6) and the other is a flavin adenine dinucleotide (FAD)-dependent polyamine oxidase (PAO, EC 1.5.3.11). PAOs, using FAD as cofactor, catalyze Spd and Spm to produce 4-aminobutanal and *N*-(3-aminopropyl)-4-aminobutanal, respectively, as well as hydrogen peroxide (H_2_O_2_) which acts as an important signaling to regulate the expression of numerous genes relative to the stress response in the back conversion (BC-type) pathway; in addition to 1,3-diaminopropane and H_2_O_2_ in the terminal catabolism (TC-type) pathway [46,47,48,49,51,52,53,54,60].

## 4. PAOs in Plants

**Figure 2:**
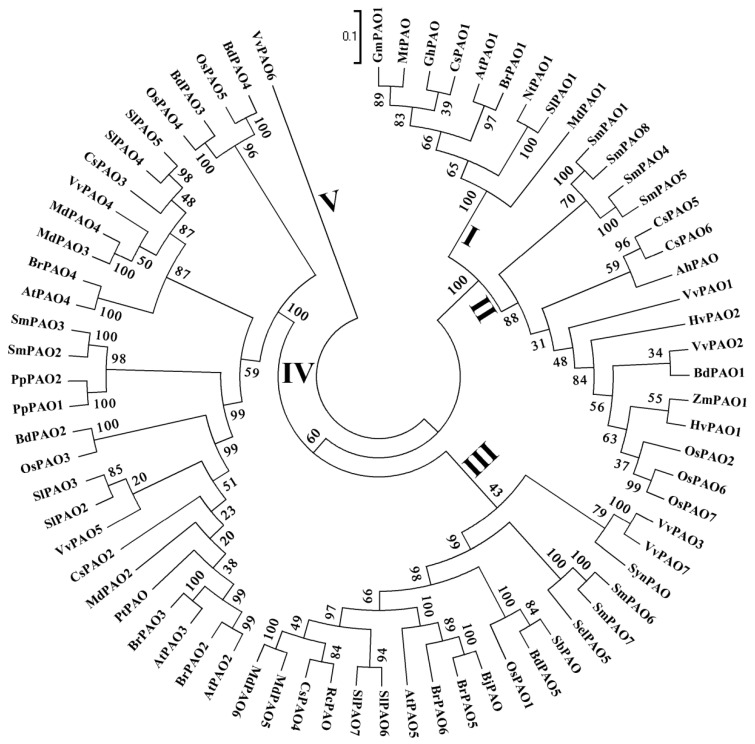
Phylogenetic relationship of polyamine oxidases (PAOs) among rice, Arabidopsis, tomato, and other plants. The neighbor-joining tree was constructed by amino acid sequence alignment using Clustal X 1.83 and MEGA 5.0. The bootstrap values, displayed at the branch nodes, were obtained with 1000 repetitions. Roman numerals (I~V) indicate clade numbers. The analyzed genes and their accession numbers are listed in Table 1. Os, *Oryza sativa*; At, *Arabidopsis thaliana*; Sl, *Solanum lycopersicum*; Bd, *Brachypodium distachyon*; Br, *Solanum lycopersicum*; Cs, *Citrus sinensis*; Sm, *Selaginella moellendorffii*; Vv, *Vitis vinifera*; Md, *Malus domestica*; Sel, *Selaginella lepidophylla*; Zm, *Zea mays*; Hv, *Hordeum vulgare*; Pp, *Physcomitrella patens*; Rc, *Ricinus communis*; Nt, *Nicotiana tabacum*; Bj, *Brassica juncea*; Pt, *Populus trichocarpa*; Sb, *Sorghum bicolor*; Gm, *Glycine max PAO1-like*; Mt, *Medicago truncatula*; Ah, *Amaranthus hypochondriacus*; Gh, *Gossypium hirsutum*; Syn, *Synechocystis*.

Up to now, more and more plant PAOs have been cloned and functionally identified. In Figure 2, we analyzed the phylogenetic relationship among seventy-three plant PAOs from twenty-four species. The plant PAOs are grouped into five clades I~V in the phylogenetic tree, as shown in Figure 2. Clade-I has nine members including Arabidopsis PAO (AtPAO1) and tomato PAO (SlPAO1) [48,61,62,63]. Clade-II contains sixteen genes including three rice PAOs (OsPAO2, OsPAO6~7) [48,60,63]. Based on previous studies, the clade II may present apoplastic PAOs that catalyze terminal oxidation reactions [36,42,44,45,46,49,55,60]. Clade-III consists of nineteen members including rice PAO (OsPAO1), Arabidopsis PAO (AtPAO5), and two tomato PAOs (SlPAO6~7) [49,51,63]. Clade-IV contains twenty-eight PAOs from eight different species including three rice PAOs (OsPAO3~5), three Arabidopsis PAOs (AtPAO2~4), and four tomato PAOs (SlPAO2~5) [12,47,48,50,61,63,64]. The clade V so far includes only a *Vitis vinifera* PAO (VvPAO6). Currently, almost all PAOs of the rice and Arabidopsis have been well determined, and we recently identified the tomato PAOs. Thus, we will review on the advance of PAOs from these three species, as well as other plant species, in this manuscript.

### 4.1. Rice PAOs

Ono et al. reported that seven *PAOs* exist in rice, orderly named as *OsPAO1*~*OsPAO7* [47]. He and his colleagues found *OsPAO3~5* are similarly and highly expressed in two-week-old seedlings and mature plants, whereas the other four *OsPAO* members are only expressed at very low levels in all tissues. Especially, *OsPAO2*, *OsPAO6,* and *OsPAO7* are expressed at almost negligible levels, as shown in Table 2 [47,49]. They also found the purified recombinant OsPAO3 strongly catalyzes Spd to Put, and also utilizes Spm, T-Spm, and Nor-Spm as substrates in vivo. The OsPAO4 and OsPAO5 proteins prefer to use Spm and T-Spm as substrates, but cannot oxidize Spd to Put, as shown in Table 2 [46,47]. The results suggested that OsPAO3 catalyzes a full BC-type pathway, while OsPAO4 and OsPAO5 only catalyze a partial BC-type pathway, as shown in Table 2 [46,47]. Besides, we found that OsPAO1, localized to the cytoplasm of onion epidermal cells, prefers to use Spm and T-Spm as substrates, and oxidizes these substrates to Spd but not to Put, as shown in Table 2 [46,48]. OsPAO1 and AtPAO5, both of which lack of intron, share high identity at the amino acid levels and exhibit quite similar predicted protein tertiary structures [50]. When the full length cDNA of OsPAO1 was fused to a constitutive promoter and subsequently transformed into the loss-of-function mutant *Atpao5-2*, the transgenic plants restored normal T-Spm sensitivity, which can grow in the presence of low levels of T-Spm; whereas the control with the introduction of *OsPAO3*—a peroxisome localized *PAO*—into *Atpao5-2* mutants did not complement the phenotype [50]. These genetic evidences indicated that *OsPAO1* and *AtPAO5* are functionally orthologous genes in Arabidopsis and rice [50].

Interestingly, our group found that OsPAO7, with high amino acid identity and very similarly predicted protein 3-D structures to ZmPAO1, which is the best characterized maize PAO catalyzed TC-type reaction, is subcellularly localized to the apoplastic space with the aid of a signal peptides (SPs, amino acid position 1-19) and transmembrane domains (TDs, amino acid position 20-29) in its N-terminal, as shown in Table 2 [46,49]. The recombinant OsPAO7 produces 1,3-diaminopropane from both Spd and Spm, indicating that OsPAO7 is the first TC-type enzyme in rice, as shown in Table 2 [46,49]. The observation of *OsPAO7_pro_:GFP* transgenic rice plants showed that *OsPAO7* is specifically expressed in anther walls and pollens with an expressional peak at the bicellular pollen stages, as shown in Table 2 [46,49]. Such results suggest that *OsPAO7* might have special roles in floral differentiation, especially in anther development and fertility, as shown in Table 2. Recently, Sagor et al. reported that the DNA sequence of the presumed coding region (accession number NM_001069545) for *OsPAO6* obtained from the National Center for Biotechnology Information (NCBI) public database is incorrect [60]. They successfully cloned the correct full-length cDNA of 1742 bp (accession number XM_015755533) by rapid amplification of the cDNA ends (RACE) in the 5′-end using 5′-RACE [60]. The correct *OsPAO6*, encoding a 497-amino acid protein, shows 92% identity and very similar protein tertiary structures to *OsPAO7*, and it is subcellularly localized to the plasma membrane, suggesting that OsPAO6 possibly also acts like OsPAO7 having the TC-type activity [46,49,60]. Furthermore, *OsPAO6* was induced by exogenous jasmonic acid, implying *OsPAO6* may be involved in stress tolerance [60]. The last rice PAO, OsPAO2, might have no enzyme activity due to a long truncation at the amino terminal [46,49,60]. However, we could not rule out the possibility that the cDNA sequence of *OsPAO2* derived from NCBI might be incorrect like the case of OsPAO7.

Up to now, the knowledge of the biological functions of *OsPAOs* remains limited. Chen et al. found that *OsPAO1~7* is most important for rice germination compared to the subfamilies’ members *OsPAO8~11* encoding histone lysine-specific demethylases, especially *OsPAO5* which probably regulates rice seed germination via PAO-generated H_2_O_2_ signaling to mediate coleorhiza-limited rice seed germination [65].

Above all, two different kinds of PAOs exist in rice; one is BC-type (OsPAO1, OsPAO3~5), the other is TC-type (OsPAO7, and OsPAO6 possibly also has this activity), as shown in Table 2 [46,49,60]. To fully understand the biological functions of *OsPAOs* in various developmental and physiological processes, molecular and genetic approaches like CRISPR/Cas9-mediated loss-of-function mutants and ubiquitin promoter enhanced overexpression transgenic plants should be generated.

### 4.2. Arabidopsis PAOs

The Arabidopsis genome contains five *PAOs*, named as *AtPAO1* to *AtPAO5*. The recombinant protein of the former four *AtPAOs*, AtPAO1~4, have been homogenously purified and characterized [12,39,61,62,64,65]; besides, AtPAO5 also has been purified and biochemically characterized [46,51]. In detail, AtPAO1, subcellularly localized in cytoplasm, catalyzes a BC-type reaction, and prefers to utilize Spm, T-Spm, and NorSpm as substrates [39]; AtPAO2~4, localized to peroxisomes, all display a BC-type reaction with different substrate specificity [12,61,62,64]. AtPAO2~3 oxidize Spm to Put in a full BC-type reaction via Spd, whereas the other peroxisomal AtPAO4 mainly catalyzes the partial BC-type because only very few Put can be detected when Spm was used as the substrate [61].

Five Arabidopsis *PAOs* showed different expression patterns. *AtPAO1* is specifically expressed in the root transition region (between the meristematic and elongation zones of the root) and anther tapetum [65], and Takahashi et al. also found that *AtPAO1* is specifically expressed in anthers [62]. *AtPAO1* was reported to be involved in environment stress tolerance [39,65], and the expression patterns imply *AtPAO1* may also play roles in root development and fertility, as shown in Table 2 [62]. *AtPAO2* is mainly expressed in the root and shoot meristematic area, the vein of rosette leaves, as well as the anthers, suggesting that *AtPAO2* might function in the development of roots, shoots, leaves, and flowers, as shown in Table 2 [62]. *AtPAO3* and *AtPAO4* display similar expression patterns, which are expressed in all tissues and whole growth stages, especially in roots, leaves, and flowers, suggesting that these two members may mediate various significant growth processes, as shown in Table 2 [62]. *pao4-1* and *pao4-2*, two independent lines of *AtPAO4* loss-of-function mutants, have 10-fold higher Spm levels compared to wild type, and delay dark-triggered senescence [66]. The last Arabidopsis PAO, *AtPAO5*, is expressed in all developmental stages, with strongest expression in roots, stems, leaves, and floral organs, as shown in Table 2 [51,62].

*AtPAO5* is a relatively completely explained Arabidopsis PAO, and its gene product AtPAO5 has been successfully characterized and its biological function also has been explored [51,67,68]. AtPAO5 can catalyze both Spm and T-Spm to Spd, but not to Put [51]. Our former colleagues Kim et al. reported that *AtPAO5* regulates stem elongation and the rosette leaves’ development, as shown in Table 2 [51,62]. Two *AtPAO5* T-DNA insertion mutants, *pao5-1* and *pao5-2*, both of which show about 2-fold higher levels of T-Spm, still maintain normal levels of Put, Spd, and Spm compared to the wild type controls [51]. The *pao5-1* and *pao5-2* mutants exhibit more rosette leaves, and shorter and fewer inflorescence stems at the two-month-old stage. Further genetic and morphology analysis suggested that *AtPAO5* plays roles in Arabidopsis growth and development through oxidizing T-Spm [46,51]. Ahou et al. found that AtPAO5 functions as an SMO/dehydrogenase [69]. *atpao5-2* and *atpao5-3*, two independent loss-of-function mutants of *AtPAO5*, show higher T-Spm contents, mediate metabolic and transcriptional reprogramming, and enhance salt-related stress tolerance [67]. *AtPAO5* also plays roles in the control of proper xylem differentiation through interplaying between auxin and cytokinins [68]. Above all, the *AtPAO5* mutant with higher T-Spm levels shows the similar phenotypes as *acl5* (*tkv*) and *bud2* mutants, which only contain very low or even zero T-Spm content [7,9,15,62,72,73]. These results explained that maintaining suitable T-Spm content is very important in plants.

Taken together, all five Arabidopsis PAOs catalyze BC-type reactions and mediate (or potentially mediate) the entire developmental processes in plants, as shown in Figure 2 and Table 2 [46], and their (especially the *AtPAO1~4*) biological functions need to be further unveiled in the future.

### 4.3. Tomato PAOs

Transgenic tomato plants overexpressing maize PAO (MPAO) exhibit tissue damage with lower chlorophyll content, lower photochemical efficiency of photosystem II (PSII), and DNA fragmentation compared to wild type, suggesting that the increased PAO activity cannot cope with the reactive oxygen species (ROS) generated by environmental factors [13]. In *S. lycopersicum cv*. Chiou, the expression of PAO peaked at ImG1 (fruits 0.5 cm in diameter) and ImG2 (fruits 1 cm in diameter) stages, suggesting PAO participates in developmental processes of the fruits, including the cell wall maturation [74]. Gémes et al. reported that sense-*ZmPAO* (*S-ZmPAO*) transgenic tomato plants have slightly larger leaf sizes and higher antioxidant enzyme activities; in contrast, the antisense-*ZmPAO* (*AS-ZmPAO*) transgenic tomato plants contain lower chlorophyll content index, smaller leaves, and less biomass, as well as an increment in Ca^2+^ when responding to salt stress [29]. The phenotypes of *S-ZmPAO* and *AS-ZmPAO* transgenic plants suggested that apoplastic PAO play important roles in plant growth and stress responses [29]. Most recently, we found that the model dicotyledons of the tomato plant (*Solanum lycopersicum*) has seven *PAO* genes in its genome, which were orderly named as *SlPAO1* to *SlPAO7* [63]. *SlPAO2~5*, sharing high identity (over 64%) of amino acid and showing quite similar genome organization and predicted tertiary structures, have similar tissue expression patterns [63]. Besides, *SlPAO2~4* are ubiquitously and highly expressed in the whole growth processes and all tissues, predominantly in anther, Br (breaker stage fruit), and Br+2 (two days post breaker stage fruit) [63], suggesting that *SlPAO2~4* may play dominant roles in all stages of growth especially in floral development and fruit maturity in tomato, as shown in Table 2 [63]. *SlPAO1* is expressed relatively lower than *SlPAO2~4* in all of the vegetative tissues and anthers [63]. What is more, *SlPAO6~7*, sharing quite similar identity of amino acid and very similar intron-exon organization and protein 3-D structures, are lowly expressed in vegetative and reproductive tissues, but had relatively higher expression in roots, stems, buds, and anthers than in the fruit [63], suggesting that these two tomato *PAOs* may mainly function in vegetative and anthesis tissues but not in fruit, as shown in Table 2. *SlPAOs* respond to abiotic stresses (heat, wound, cold, drought, and salt), oxygen species (H_2_O_2_ and methylviologen), phytohormones (IAA, 6-BA, GA, ABA, Eth, SA, and JA), as well as PAs (Put, Spd, Spm, and T-Spm), implying that tomato *PAOs* possibly have various functions in stress tolerances, as shown in Table 2 [63]. Taken together, *SlPAOs* possibly play vital roles in different tissues and developmental stages, especially in floral development and fruit repining. To better explain the mechanism of polyamine catabolism and biological roles of *SlPAOs*, more biochemical and genetic experiments are required.

### 4.4. PAOs in Other Plants

Recently, besides these three model plants (rice, Arabidopsis, and tomato), some other plant species have also been studied on PAO catabolism, and PAO biological functions. Plant PAOs play important roles in various stress tolerance and the programmed cell death (PCD) events through mediating H_2_O_2_ signaling which is generated by stress-induced PAO activity leading to Spd, Spm, and T-Spm oxidation [13,33,75,76,77,78,79,80,81,82,83,84,85,86,87,88,89,90,91,92,93,94,95,96,97,98]. Hatmi et al. reported that the grapevine PAO and CuAO activities were upregulated by osmotic stress and *Botrytis cinerea* infection, suggesting that PA back-conversion and/or terminal catabolism were involved in PA homeostasis under stress conditions [97]. In addition, the PAO activity increment and proline accumulation were involved in cold tolerance in *Medicago falcate* [75,76], suggesting that PAOs and proline interplay in the process of various stress responses [75,76,99,100,101]. What is more, in *salinity tolerance 1* (*st1*), a wheat salinity-tolerant line, the expression of *PAO* genes showed high expression levels, suggesting that *PAO* genes may have important functions in salinity tolerance [102].

Previously, Sagor and his colleagues reported that SelPAO5 from *Selaginella lepidophylla* back-converts Spm and T-Spm to Spd and Nor-Spd, respectively [53]. It is different from AtPAO5 and OsPAO1 which prefer to use the same substrates as SelPAO5, but both of these two enzymes convert the substrates to Spd, though three of them are from the same clade in the phylogenic relationship tree, suggesting that SelPAO5 oxidizes T-Spm at different carbon positions [53]. Most recently, they further found that the SelPAO5 can complement the dwarf phenotype of *Atpao5*, with the reduction of T-Spm content to almost normal levels of wild type, which strengthens the claim that T-Spm homeostasis is required for plant development and growth [103]. Besides, Wang and Liu firstly identified PAOs from sweet orange (*Citrus sinensis*); their results indicated that six PAO genes (*CsPAO1*–*CsPAO6*) exist in sweet orange, and they also found that CsPAO3 may have potential roles in PA back conversion in plants, while CsPAO4 catalyzes Spd and Spm as substrates for terminal catabolism [55,71]. The transgenic plants overexpressing *CsPAO4* showed growth inhibition under salt stress caused by the elevation of H_2_O_2_ which leads to oxidative damages [55]. What is more, Brikis et al. found that the expression of *MdPAO2* was obviously upregulated in apple fruit by elevating the CO_2_ concentrations under low-temperature/low-O_2_ storage for up to sixteen weeks, suggesting that *MdPAO2* is involved in respiratory activities in apple fruit storage under multiple abiotic stresses [104]. Furthermore, Takahashi et al. characterized the molecular and biochemical features of five PAOs (BdPAO1 to BdPAO5) from *Brachypodium distachyon*, and they found that BdPAO2 and BdPAO4 possibly are localized to peroxisomes [70]. Additionally, they also found that BdPAO2 catalyzes a full-back conversion pathway, and the favorite substrates of BdPAO2 and BdPAO3 are Spd and Spm, respectively [70].

Plant PAOs play significant roles in metal toxicity tolerance. Aluminum (Al), copper (Cu), and cadmium (Cd), etc. are phytotoxic to plants at high concentrations [33,81,97,105,106]. In wheat, the cell wall-bound PAO (CW-PAO) oxidized Spd and generated H_2_O_2_ under Al toxicity; in contrast, the CW-PAO activity was markedly inhibited by Put application, and subsequently reduced H_2_O_2_ accumulation in roots under Al stress, suggesting that Put plays an important protective role against Al-induced oxidative stress via inhibiting the PAO activity with lower H_2_O_2_ production [33]. Similarly, the PAO activity was enhanced by higher Cu or Cd concentrations leading to accelerating the PA back-conversion or terminal catabolism, which may be related to functionality of defense mechanisms [105,106]. To entirely understand the functional mechanism of PAOs on metal toxicity tolerance, more attractive and systematic studies are required.

Plant PAOs have important roles in plant growth and development. Around fifteen years ago, the functions of the maize PAO were investigated by the Rea group and the Cona group separately, and they found that the maize PAO plays roles in cell-wall maturation and root differentiation by producing H_2_O_2_ [107,108]. Gomez-Jimenez et al. reported that PAO and DAO have significant functions in olive fruit abscission zone (AZ) development through providing apoplastic H_2_O_2_ for cell-wall strengthening and lignosuberization events, and the peroxidase substrate is provided in these cells throughout AZ development [109]. Moreover, Rodríguez et al. reported that the increased PAO activity produces more H_2_O_2_ to generate ·O^2-^ through enhanced substrate availability and subsequently maintain maize leaf elongation under saline stress [16]. What is more, the tomato PAO is involved in vascular development via mediating H_2_O_2_ which is required by vascular differentiation and the process of polymerization of lignin precursors into lignin [110]. *Atpao3*, a loss-of-function mutant of AtPAO3 which oxidizes Spd in peroxisomes [12], shows reduced pollen tube and seed setting caused by significantly disrupted Spd-induced Ca^2+^ currents [111]. Furthermore, Agudelo-Romero et al. found that the activities of PAO and DAO are significantly increased during grape ripening, implying an important role of polyamines’ catabolism in fruit ripening [112].

### 4.5. Peroxisomal PAOs in Plants

**Figure 3:**
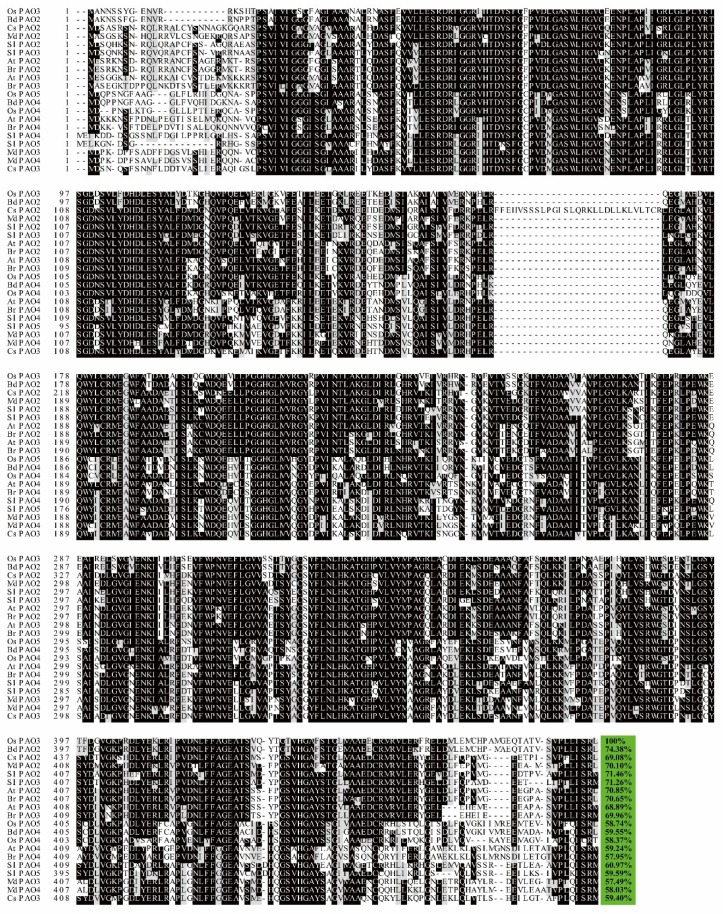
Alignment of amino acid sequences of twenty reported peroxisomal PAOs from *Oryza sativa*, *Arabidopsis thaliana*, *Solanum lycopersicum*, *Brachypodium distachyon*, *Brassica rapa*, *Citrus sinensis,* and *Malus domestica*. The alignment was performed by the Clustal X 1.83 software and exhibited by the Boxshade program (http://www.ch.embnet.org/software/BOX_form.html.). Black and gray indicate the complete and partial homology of the amino acid sequences, respectively. The percentages at the end of the alignment showed the identity between OsPAO3 and other PAOs.

In Arabidopsis, *AtPAO2~4* were speculated to be localized to peroxisomes [12,39,62,64,65]; additionally, in rice, we also found that *OsPAO3~5* are situated in peroxisomes [46,47]. Besides, recently some other groups reported that *BdPAO2* and *BdPAO4* from *Brachypodium distachyon* [70], *BrPAO2~4* from *Brassica rapa* [81], *CsPAO2~3* from *Citrus sinensis* [71], and *SlPAO2~4* from tomato [63] were predicted to be peroxisomal PAOs. All of these genes’ products classifying into clade IV, as shown in Figure 2, contain peroxisomal-targeting signals in their C-terminal, resulting in localization to peroxisome, as shown in Figure 3 [12,39,46,47,62,63,64,65,70,81]. In the apple genome, six putative apple PAO genes were identified [104]. The *MdPAO2~4* were predicted to localize in peroxisomes, whereas *MdPAO1* and *MdPAO5~6* were predicted to be cytosolic proteins [104]. In addition, four CuAO-like genes from *Arabidopsis* have two different localizations; the AtCuAO2 and AtCuAO3 are localized to peroxisomes, while the AtAO1 and AtCuAO1 are localized to apoplast [113].

**Figure 4:**
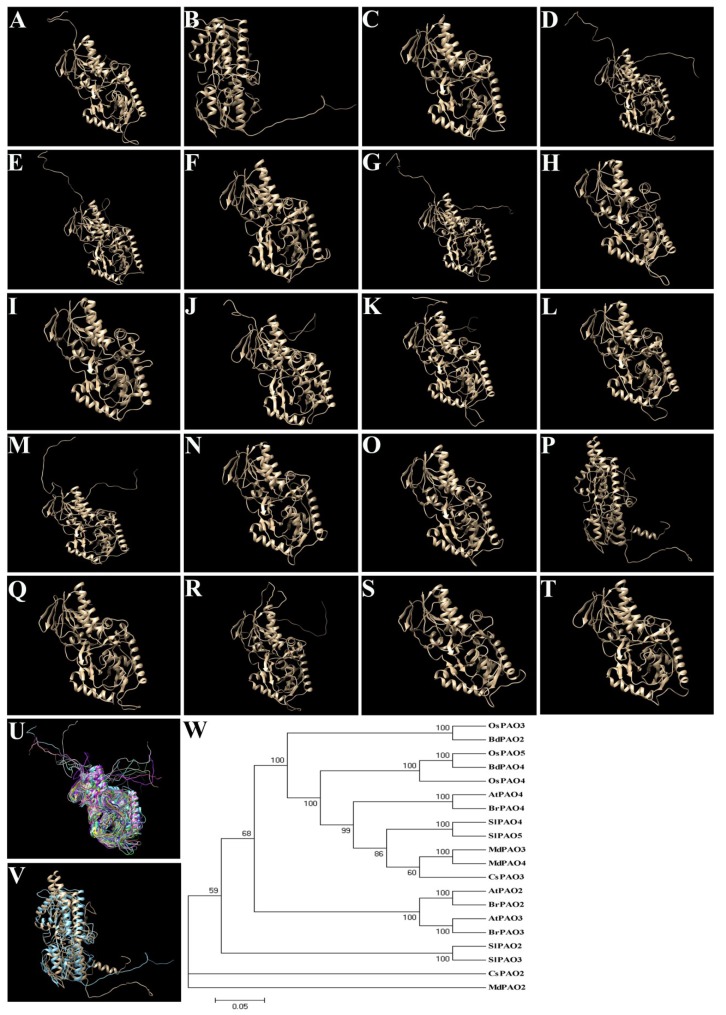
Predicted tertiary structures of the reported peroxisomal PAOs. Twenty have been reported; peroxisomal plant PAOs were analyzed. (**A**–**J**), The protein 3-D structures of OsPAO3 (**A**); OsPAO4 (**B**); OsPAO5 (**C**); AtPAO2 (**D**); AtPAO3 (**E**); AtPAO5 (**F**); SlPAO2 (**G**); SlPAO3 (**H**); SlPAO4 (**I**); SlPAO5 (**J**); BdPAO2 (**K**); BdPAO4 (**L**); BrPAO2 (**M**); BrPAO3 (**N**); BrPAO4 (**O**); CsPAO2 (**P**); CsPAO3 (**Q**); MdPAO2 (**R**); MdPAO3 (**S**); and MdPAO4 (**T**) were obtained using the Protein Structure Prediction Server program (http://ps2v3.life.nctu.edu.tw/) and Chimera 1.13 software. (**U**) Merged image of all PAOs, except OsPAO4 and CsPAO2, was performed by Chimera 1.13 software. (**V**) Merged image of OsPAO4 and CsPAO2 was similarly performed. The light blue and light yellow colors indicate the protein structures of OsPAO4 and CsPAO2, respectively. (**W**) Evolution relationship among the peroxisomal PAOs.

These peroxisomal PAOs shared high identity (over 57% compared to OsPAO3 which was set as 100%), as shown in Figure 3, and displayed quite similar predicted protein tertiary structures, as shown in Figure 4A–Q, even though these PAOs are from six different species. Interestingly, the predicted protein tertiary structures of these twenty peroxisomal PAOs almost fully merged with each other, as shown in Figure 4U, except OsPAO4 and CsPAO2 that cannot merge with other PAOs; whereas, to our surprise, the OsPAO4 and CsPAO2 were largely merged, as shown in Figure 4V. Besides, the protein sequence of CsPAO2 contains an additional twenty-nine amino acid sequence in the conserved region compared to other peroxisomal PAOs, as shown in Figure 3, that may be because of the mRNA alternative splicing, though the possible function of this additional sequence remains totally unknown. The results of phylogenetic relationship analysis, as shown in Figure 4W, also indicated that these peroxisomal PAOs are highly conserved and extremely close during evolution in the plant kingdom.

It is suggested that the peroxisomal PAOs possibly play significant roles in plant growth processes, especially in floral development, as shown in Table 2. To explore the physiological and biological significance of peroxisomal PAOs, genetic and morphological approaches are required via generating functional knock-down (or knock-out) mutants. Besides, apoplastic PAOs were found in monocotyledonous plants such as maize PAO (ZmPAO), barley PAO (HvPAOs), and rice PAO (OsPAOs), which were involved in TC-type pathways to catalyze PA terminal oxidation [36,42,44,45,46,49,60,107]. In dicots, apoplastic PAOs may be present in limited species [55]. What is more, the cytoplasmic PAOs were characterized in Arabidopsis (AtPAO1 and AtPAO5) [39,51,61,62,65] and rice (OsPAO1) [46,48], which catalyzed PA back conversion reactions. However, the roles of the three types of PAOs in plant growth and development, and stress tolerance through PA homeostasis and/or H_2_O_2_ generation, remain fragmentary. Thereby, the significance of the functional difference between peroxisomal or cytoplasmic PAOs and apoplastic PAOs remains to be clarified and should be addressed in future work.

## 5. Conclusions and Future Perspective of *PAOs* Research in Plants

**Figure 5:**
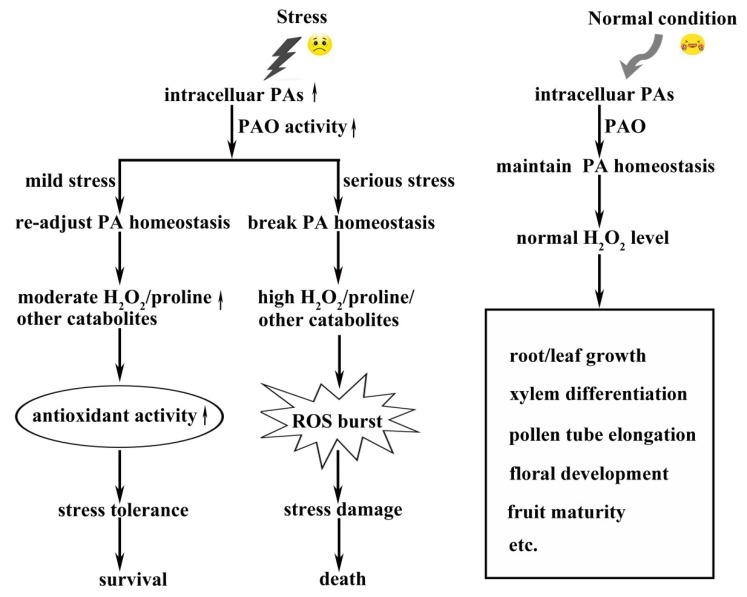
Diagrammatic representation of the roles of PAO involved in developmental growth and environmental stress response in plants. The thick upright arrows indicate increase in the activity or concentrations. The cartoon pictures of smiling and bitter faces indicate the plant growth under normal or stress conditions, respectively. ROS: reactive oxygen species.

In the past years, some PAO genes were cloned and functionally identified from different plant species. Some research groups focus on the PA catabolism pathway, meanwhile, more and more researchers pay intense attention to the biological roles of PAOs. As it is known, when plants grow under normal conditions, the intracellular PAs maintain homeostasis, and the normal level of H_2_O_2_ is generated by PAOs. Subsequently the H_2_O_2_ signal participates in the developmental processes such as root growth, xylem differentiation, pollen tube growth, fruit development, etc., as shown in Figure 5 [65,69,99,111,112]. However, the PAs homeostasis might encounter challenges under stress conditions. The enhanced accumulation of stress-induced PAs requires higher PAO activity to rebalance the PAs homeostasis. If just under mild stress, the plants can overcome the unpleasant period via the antioxidant reaction with the aid of proline and other catabolites that were also induced by stress [76,99,100,101]. If under severe stress and longtime stress conditions, the PAO activity markedly increases to reduce the stress-induced intracellular PAs level with high H_2_O_2_ accumulation, leading to a ROS burst which may result in death, as shown in Figure 5. The antioxidant activity cannot offset the strong ROS burst, though the levels of proline and other catabolites are also upregulated by stress, as shown in Figure 5 [93,94].

Recently, the enzyme features of all the Arabidopsis *PAOs* and most rice *PAOs* have been identified, but their biological roles remain largely unclear. Meanwhile, the tomato *PAOs* have been cloned, but its catabolic activities and biological functions are still unknown. What is more, the exact roles of the highly conserved peroxisomal *PAOs* in plants are still fuzzy. Furthermore, why does rice have two different types of *PAO* catabolic pathways (the BC-type and TC-type pathway)? In addition, the exact mechanism of PA metabolism and the PA-cycle—PA exodus—as well as the possible ratio between the back-conversion and terminal catabolism in plants needs to be uncovered. Finally, what is the possible relationship between PAO and proline when plants fight against environmental stresses? To fully understand the roles of *PAOs* in plant development and stress interactions, intensive studies are required via generating loss-of-function mutants and overexpression transgenic plants which will greatly help further explore the biochemical and physiological roles of these *PAOs*.

## Figures and Tables

**Table 1:** List of the accession numbers of the plant PAOs used in Figure 2.

Gene Name	Accession No.	Gene Name	Accession No.	Gene Name	Accession No.	Gene Name	Accession No.
*OsPAO1*	NM_001050573	*BdPAO1*	XM_003573843	*SmPAO3*	XP_002968082.1	*PpPAO2*	XM_001776435
*OsPAO2*	NM_001055782	*BdPAO2*	XM_010242147	*SmPAO4*	XP_002969966.1	*RcPAO*	XM_002521542
*OsPAO3*	NM_001060458	*BdPAO3*	XM_003580746	*SmPAO5*	XP_002981437.1	*PtPAO*	XM_002306729
*OsPAO4*	NM_001060753	*BdPAO4*	XM_003580747	*SmPAO6*	XP_002984796.1	*SbPAO*	XM_002448510
*OsPAO5*	NM_001060754	*BdPAO5*	XM_003566997	*SmPAO7*	XP_002985859.1	*GmPAO1*	XP_003535841.1
*OsPAO6*	XM_015755533	*BrPAO1*	Bra006210	*SmPAO8*	XP_002986593.1	*MtPAO*	XP_003599417.1
*OsPAO7*	NM_001069546	*BrPAO2*	Bra037741	*VvPAO1*	VIT_01s0127g00750	*SynPAO*	WP_011153630.1
*AtPAO1*	NM_121373	*BrPAO3*	Bra003362	*VvPAO2*	VIT_01s0127g00800	*PpPAO1*	XM_001756812
*AtPAO2*	AF364952	*BrPAO4*	Bra039742	*VvPAO3*	VIT_03s0017g01000	*ZmPAO1*	NM_001111636
*AtPAO3*	AY143905	*BrPAO5*	Bra011132	*VvPAO4*	VIT_04s0043g00220	*AhPAO*	AAM43922.1
*AtPAO4*	AF364953	*BrPAO6*	Bra024137	*VvPAO5*	VIT_12s0028g01120	*GhPAO*	KC762210.1
*AtPAO5*	AK118203	*CsPAO1*	Cs7g02060.1	*VvPAO6*	VIT_12s0055g00480	*HvPAO1*	AJ298131
*SlPAO1*	XP_004229651	*CsPAO2*	Cs7g18840.2	*VvPAO7*	VIT_13s0019g04820	*HvPAO2*	AJ298132
*SlPAO2*	XP_004243630	*CsPAO3*	Cs6g15870.1	*MdPAO1*	ANJ77637.1	*SelPAO5*	LC036642
*SlPAO3*	XP_004251556	*CsPAO4*	Cs4g14150.1	*MdPAO2*	ANJ77639.1	*NtPAO*	AB200262
*SlPAO4*	XP_004232664	*CsPAO5*	Cs7g23790.1	*MdPAO3*	ANJ77642.1	*BjPAO*	AY188087
*SlPAO5*	XP_004234492	*CsPAO6*	Cs7g23760.1	*MdPAO4*	ANJ77638.1		
*SlPAO6*	XP_004243758	*SmPAO1*	XP_002965265.1	*MdPAO5*	ANJ77640.1		
*SlPAO7*	XP_004239292	*SmPAO2*	XP_002965599.1	*MdPAO6*	ANJ77641.1		

**Table 2:** Summary of PAOs in rice, Arabidopsis, and tomato.

Gene Name	Gene ID	Subcellular Localization	Substrate Specificity	Mode of Reaction	Tissue Expression	Functions (or Potential Functions)	Reference
***Oryza sativa***							
*OsPAO1*	Os01g0710200	cytoplasm	Spm, T-Spm	BC	rachis	rachis development, tolerances, seed germination	[31,46,47,48]
*OsPAO2*	Os03g0193400	n.d.	n.d.	n.d.	root (with very low expression levels)	tolerances, seed germination	[31,46,49]
*OsPAO3*	Os04g0623300	peroxisome	Spd, Spm, T-Spm	BC	All stages. Strongest expressed in leaf, rachis, node, lower leaf blade, mature floral organ	leaf and node development, floral development, fertility, seed germination	[31,46,47]
*OsPAO4*	Os04g0671200	peroxisome	Spm, T-Spm	BC	rachis, mature floral organ	rachis and floral development, fertility, seed germination	[31,46,47]
*OsPAO5*	Os04g0671300	peroxisome	Spm, T-Spm	BC	flag leaf, lower leaf blade, leaf sheath, mature floral organ	development of leaf and flower, fertility, seed germination	[31,46,47]
*OsPAO6*	Os09g0368200	apoplast	n.d.	TC (?)	expressed at negligible levels	tolerances, seed germination	[31,46,60]
*OsPAO7*	Os09g0368500	apoplast	Spm, Spd	TC	anther, pollen	floral development, fertility, seed germination	[31,46,49]
***Arabidopsis thaliana***							
*AtPAO1*	At5g13700	cytoplasm	Spm, T-Spm	BC	root transition region, anther	stress tolerance, root development, fertility	[39,46,61,62,65]
*AtPAO2*	At2g43020	peroxisome	Spd, Spm, T-Spm	BC	root meristem, anther, main vein of rosette leaf	root development, fertility, vein development of leaf	[46,61,62,64,65]
*AtPAO3*	At3g59050	peroxisome	Spd, Spm, T-Spm	BC	All stages. Strongest expressed in root tip, flower, guard cell	root and leaf development, fertility	[12,46,61,62,65]
*AtPAO4*	At1g65840	peroxisome	Spm, T-Spm	BC	All stages. Strongest expressed in root and floral organ	Delay dark-induced senescence. Root development, fertility	[46,61,62,64,65,66]
*AtPAO5*	At4g29720	cytoplasm	Spm, T-Spm	BC	All stages. Strongest expressed in mature leaf, vascular tissue, flower, stem	xylem differentiation, stem elongation, development of rosette leaves and vein, tolerance	[46,51,61,62,65,67,68,69]
***Solanum lycopersicum***							
*SlPAO1*	Solyc01g087590	n.d.	n.d.	n.d.	root, stem, leaf of seedling stage	vegetative growth	[63]
*SlPAO2*	Solyc07g043590	peroxisome (?)	n.d.	n.d.	All stages. Strongest expressed in anther, Br, Br+2, stem	floral development, fruit maturity	[63]
*SlPAO3*	Solyc12g006370	peroxisome (?)	n.d.	n.d.	All stages. Strongest expressed in anther, Br, Br+2, leaf	floral development, fruit maturity	[63]
*SlPAO4*	Solyc02g081390	peroxisome (?)	n.d.	n.d.	All stages. Strongest expressed in anther, Br, Br+2, Br+7, root, leaf	floral development, fruit maturity	[63]
*SlPAO5*	Solyc03g031880	peroxisome (?)	n.d.	n.d.	All stages. Strongest expressed in anther, leaf, stem	floral development	[63]
*SlPAO6*	Solyc07g039310	n.d.	n.d.	n.d.	root, stem of seedling stage	vegetative growth	[63]
*SlPAO7*	Solyc05g018880	n.d.	n.d.	n.d.	root, stem of seedling stage	vegetative growth	[63]
***Brachypodium distachyon***							
*B* *dPAO1*	XM_003573843	n.d.	n.d.	n.d.	expressed at very low levels	unknown	[70]
*B* *dPAO2*	XM_010242147	peroxisome (?)	Spd, Spm, T-Spm, Nor-Spm, Nor-Spd	BC	All stages. Highly expressed in leaf, stem, and inflorescence	development of stem and inflorescence	[70]
*B* *dPAO3*	XM_003580746	n.d.	Spm,	BC	leaf, stem, and inflorescence	development of stem and inflorescence	[70]
*B* *dPAO4*	XM_003580747	peroxisome (?)	n.d.	n.d.	leaf, stem, and inflorescence	development of stem and inflorescence	[70]
*B* *dPAO5*	XM_003566997	n.d.	n.d.	n.d.	expressed at very low levels	unknown	[70]
***Citrus sinensis***							
*CsPAO1*	Cs7g02060.1	n.d.	n.d.	BC (?)	leaf, stem, root, cotyledon	root growth, vegetative growth	[55,71]
*CsPAO2*	Cs7g18840.2	peroxisome (?)	n.d.	BC (?)	leaf, stem, root, cotyledon	root growth, vegetative growth	[55,71]
*CsPAO3*	Cs6g15870.1	peroxisome (?)	n.d.	BC (?)	leaf, stem, root, cotyledon	root growth, vegetative growth	[55,71]
*CsPAO4*	Cs4g14150.1	apoplast	Spd, Spm	TC	leaf, stem, root	seed germination, the growth of root and vegetative, salt tolerance	[55,71]
*CsPAO5*	Cs7g23790.1	n.d.	n.d.	BC (?)	leaf, stem, root, cotyledon	root growth, vegetative growth	[55,71]
*CsPAO6*	Cs7g23760.1	n.d.	n.d.	BC (?)	stem, root, cotyledon	root growth, vegetative growth	[55,71]

n. d., not determined; Br, breaker stage fruit; Br+2, two days post breaker stage fruit; Br+7, seven days post breaker stage fruit; BC, back conversion; TC, terminal catabolism.
